# Withaferin A Alters Intermediate Filament Organization, Cell Shape and Behavior

**DOI:** 10.1371/journal.pone.0039065

**Published:** 2012-06-15

**Authors:** Boris Grin, Saleemulla Mahammad, Tatjana Wedig, Megan M. Cleland, Lester Tsai, Harald Herrmann, Robert D. Goldman

**Affiliations:** 1 Department of Cell and Molecular Biology, Feinberg School of Medicine at Northwestern University, Chicago, Illinois, United States of America; 2 Division Biophysics of Macromolecules, German Cancer Research Center, Heidelberg, Germany; Dalhousie University, Canada

## Abstract

Withaferin A (WFA) is a steroidal lactone present in *Withania somnifera* which has been shown *in vitro* to bind to the intermediate filament protein, vimentin. Based upon its affinity for vimentin, it has been proposed that WFA can be used as an anti-tumor agent to target metastatic cells which up-regulate vimentin expression. We show that WFA treatment of human fibroblasts rapidly reorganizes vimentin intermediate filaments (VIF) into a perinuclear aggregate. This reorganization is dose dependent and is accompanied by a change in cell shape, decreased motility and an increase in vimentin phosphorylation at serine-38. Furthermore, vimentin lacking cysteine-328, the proposed WFA binding site, remains sensitive to WFA demonstrating that this site is not required for its cellular effects. Using analytical ultracentrifugation, viscometry, electron microscopy and sedimentation assays we show that WFA has no effect on VIF assembly *in vitro*. Furthermore, WFA is not specific for vimentin as it disrupts the cellular organization and induces perinuclear aggregates of several other IF networks comprised of peripherin, neurofilament-triplet protein, and keratin. In cells co-expressing keratin IF and VIF, the former are significantly less sensitive to WFA with respect to inducing perinuclear aggregates. The organization of microtubules and actin/microfilaments is also affected by WFA. Microtubules become wavier and sparser and the number of stress fibers appears to increase. Following 24 hrs of exposure to doses of WFA that alter VIF organization and motility, cells undergo apoptosis. Lower doses of the drug do not kill cells but cause them to senesce. In light of our findings that WFA affects multiple IF systems, which are expressed in many tissues of the body, caution is warranted in its use as an anti-cancer agent, since it may have debilitating organism-wide effects.

## Introduction

Withaferin A (WFA) is a steroidal lactone isolated from *Withania somnifera* a plant that has been used in Indian (Ayurvedic) medicine for centuries. WFA has been employed in the treatment of a wide range of diseases including skin disease, diabetes, arthritis, and epilepsy [1]. Recently, it has been shown that WFA binds to and alters the distribution of vimentin intermediate filaments (VIF) in cultured endothelial cells [2]. Biochemical studies using tryptic fragments and molecular modeling suggest that WFA binds covalently to cysteine-328 in the helix termination or 2B region of the α-helical central rod domain of vimentin [2]. Based upon these findings, it has been proposed that WFA might be useful as an anti-tumor agent since vimentin expression is frequently up-regulated as cancer cells undergo the epithelial to mesenchymal transition (EMT) associated with metastasis [3]. Thus WFA may specifically target metastatic cancer cells [4]. This possibility has been supported by the finding that WFA inhibits the growth of and induces apoptosis in cells derived from several human cancers including pancreatic carcinoma (Panc-1, MIA-PaCa2, BXPC3), osteosarcoma (Saos-2), leukemia, and lymphoma [5]–[7]. WFA also inhibits the growth and metastasis of tumors in mouse models of soft tissue sarcoma, as well as breast and pancreatic cancer [4], [7], [8]. The role of WFA in decreasing the size and spread of tumors may be related to its ability to inhibit angiogenesis [2], [8], [9]. However, little is known about the specific effects of WFA on vimentin either at the cellular or biochemical levels.

Vimentin is a member of the large family of IF proteins which are encoded by more than seventy genes [10]. The expression of these genes has been shown to be developmentally regulated in a cell type- and tissue-specific manner. Intermediate filaments are categorized into five or six types based on their amino acid sequence homologies [11]. For example, vimentin is a type III IF protein typically expressed in cells that originate from the mesenchyme (fibroblasts, immune and endothelial cells). And the types I and II IF proteins, the keratins, are typically found in epithelial cells. Regardless of their tissue origin, the defining feature of all IF proteins is a central highly conserved α-helical rod domain. Within the central rod, the N-terminal 1A helix initiation and the C-terminal 2B helix termination domains are the most highly conserved. These domains are essential for the proper assembly of IF proteins such as vimentin into mature 10 nm diameter filaments. Within cells, IF proteins assemble in a series of steps involving different organizational states. In the case of vimentin, it first assembles into non-filamentous particles that polymerize into short IF (squiggles), which in turn anneal end-to-end to form long IF [12]. There is evidence that the regulation of these different assembly states involves numerous kinases. These include PKA [13], Akt [14], and Cdk1 [15] which phosphorylate vimentin at one or more of over 40 known or potential sites, and in some cases alter the organization and assembly state of VIF [16]–[18]. Additionally, VIF interact in a complex manner with the two other major cytoskeletal systems, microtubules and microfilaments. As a result the normal, dispersed organization of VIF has been shown to be dependent on the presence of microtubules and microtubule-based motors, the kinesins and conventional dynein [19]. VIF also interact with actin stress fibers [20], [21] via IF associated proteins such as plectin [22], [23].

It has been shown that a normal cytoplasmic organization of VIF plays an important role in determining the shape and motility of mesenchymal cells such as fibroblasts. For example, the induction of vimentin expression and its assembly into VIF in MCF7 breast epithelial cells, which normally express only keratin IF, causes a rapid change to a mesenchymal cell shape. This shape transition is accompanied by a dramatic increase in cell motility, thereby mimicking the behavioral changes seen during the EMT [24]. Furthermore, the specific role of VIF in cell motility has recently been shown to be related to the formation and positioning of lamellipodia at the leading edge of migrating fibroblasts [25].

Since there are no known small molecule inhibitors that specifically interfere with IF structure and function, it is important to determine how WFA interacts with VIF and to determine the specificity of these interactions with respect to altering VIF functions. This is especially important as WFA has recently been proposed as an anti-tumor agent. Even though a number of recent papers have reported the use of WFA as an inhibitor of VIF function, little is known regarding the mechanisms responsible for its effects on IF or the alterations in cell physiology that accompany WFA treatment. In this study we investigate the effects of short and long-term WFA treatment on VIF and its effects on cell shape, motility and proliferation. We have also determined the specificity of WFA for VIF by testing its affects on other types of IF, such as those comprised of the keratins, peripherin, and neurofilaments, as well as the microtubule and microfilament cytoskeletal systems.

## Results

### Withaferin A Induces the Reorganization of Vimentin Intermediate Filaments

**Figure 1:**
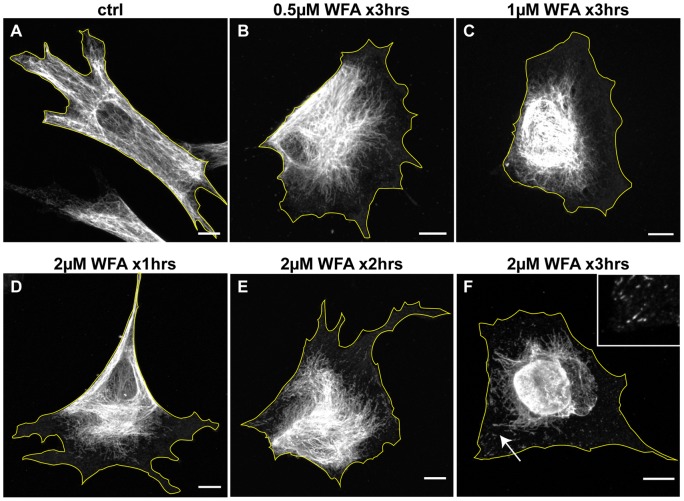
WFA treatment alters the subcellular organization of VIF. BJ-5ta cells were treated for 3 hrs (A–C and F) with DMSO alone (A), 0.5 μM WFA (B), 1 μM WFA (C), and 2 μM WFA (F). In addition, cells treated with 2 μM WFA are depicted after 1 hr (D) and 2 hrs (E) which show that the changes in VIF organization take place gradually. Cells were fixed and processed for immunofluorescence with vimentin antibodies. Arrow: a region depicted at higher magnification in the inset, showing non-filamentous vimentin particles and short IF or squiggles. Scale bars =10 μm.

**Figure 2:**
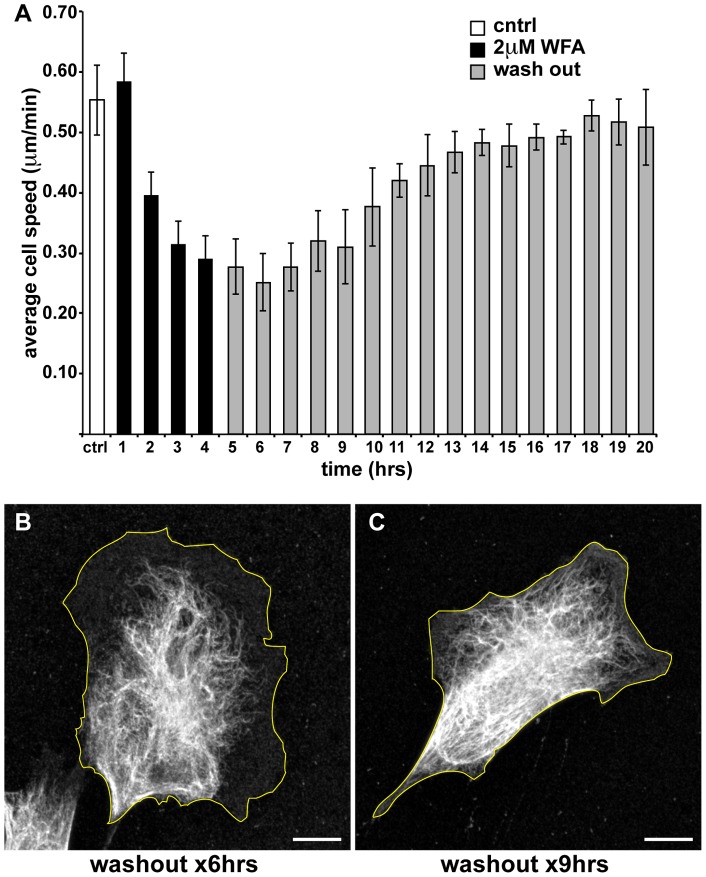
WFA treatment inhibits cell motility. (A) The average speed of BJ-5ta fibroblasts was calculated before treatment (white bar), during incubation with 2 μM WFA for 4 hrs (black bars) and after the cells were allowed to recover in fresh medium (gray bars). (B and C) Cells were treated with 2 μM WFA for 3 hrs and then placed into fresh medium followed by fixation and processing for immunofluorescence with vimentin antibodies after 6h rs (B) and 9 hrs (C). Scale bars =10 μm.

The effects of withaferin A (WFA) on vimentin IF (VIF) networks were determined in human fibroblasts (BJ-5ta) by immunofluorescence. In controls, VIF networks extend throughout the cytoplasm, from the nucleus to peripheral regions (Fig. 1A). After a three-hour exposure to WFA, VIF networks retract from the cell periphery towards the nucleus. The extent of retraction varies with the concentration of WFA. After 3 hr in 0.5 μM WFA, the retraction is less extensive (Fig. 1B) when compared to 1.0 μM WFA, which causes more extensive retraction (Fig. 1C). Eighty-two percent (82±1.04%, n=315) of cells treated with 2 μM WFA show an extensive reorganization of VIF into the perinuclear region after 3 hr. In addition, a significant number of non-filamentous vimentin particles and short IF or squiggles, both precursors to long IF, are observed between the juxtanuclear aggregates and the cell surface [12]. These latter structures which represent normal intermediate steps in the disassembly/assembly of mature VIF are significantly more visible following WFA treatment. In addition, the reorganization of VIF involves several intermediate stages of retraction seen at one and two hours following exposure to 2 μM WFA, prior to the formation of the large perinuclear aggregates (Fig. 1D–F). The effects of WFA treatment are reversible following 3 hr of exposure. The normal organization of the VIF network is gradually re-established in the majority of cells within 6 to 9 hrs following removal of WFA from the cell culture medium (Fig. 2 B, C). We use 2 μM WFA in BJ-5ta cells throughout the rest of this study, unless otherwise noted, as it has a maximal reversible impact on VIF organization over short time periods.

### Withaferin A Alters Cell Shape and Motility

Previous studies from our laboratory have demonstrated that changes in the organization of VIF networks within fibroblasts affect cell shape, motility and the formation of lamellipodia [24]–[26]. Therefore, we determined whether the changes in VIF organization induced by WFA treatment alter cell shape by using form factor analysis, a measure of the roundness of a cell [27]. The form factor of BJ-5ta fibroblasts treated with 2 μM WFA is 0.39±02 (n=150) after 3 hrs, which is significantly greater than controls (0.29±.01; n=100, p<0.05). Cells treated with 0.5 μM and 1 μM WFA also show statistically significant (p<0.05) increases in form factor compared to controls, 0.36±02 (n=57) and 0.35±.02 (n=50), respectively. These changes in form factor demonstrate a change from the typical asymmetric elongated shapes of fibroblasts to the more rounded shapes of epithelial cells [24].

The effects of WFA on BJ-5ta motility were determined using time-lapse imaging. The average rate of cell migration is significantly slower during the first 240 min following the addition of 2 μM WFA (0.59±05 μm/min, 0.42±04 μm/min, and 0.31±04 μm/min and 0.29±04 μm/min; after one, two, three, and four hours, respectively; Fig. 2A) compared to control cells (0.56±06 μm/min). Upon removal of WFA after 180 min, normal motility is re-established to pre-treatment levels within 9 hrs, which corresponds to the time of the re-establishment of normal fibroblastic shape and normal VIF organization (Fig. 2B, C). The decrease in the rate of motility also correlates well with the extent of VIF network retraction towards the cell nucleus (Fig. 1D–F).

**Figure 3:**
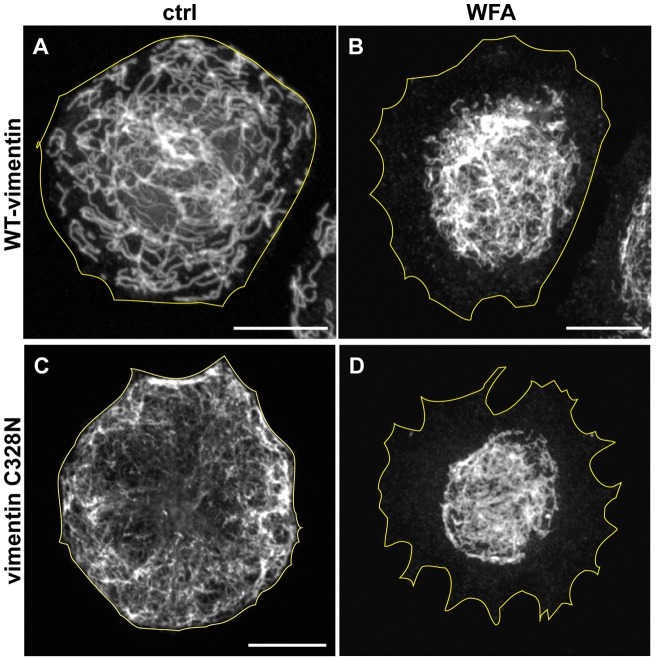
Cysteine-328 is not required for the effects of WFA on VIF. SW13-1HF5 cells which are null for cytoplasmic IF, were transfected with wild-type vimentin (A) and vimentin C328N (C). The cells were then treated for 3 hrs with DMSO (A and C) or 9 μM WFA (B and D). Scale bars =10 μm.

As described above, there is evidence that WFA binds covalently to vimentin's only cysteine residue (cys328), which is located in the 2B region of the central rod domain [2]. Therefore, we reasoned that an excellent control for our experiments would be the expression of a cysteine-null vimentin. Cysteine-328 was converted to asparagine (vimC328N) in the vimentin cDNA. This mutant construct was transfected into SW13-1HF5 cells which are null for vimentin and do not express other types of cytoskeletal IF. Within 24 hrs following transfection, the mutant protein assembles into IF networks that are indistinguishable from those assembled from wild-type vimentin (Fig. 3A, C). Upon incubation of these transfected cells with 9 μM WFA (the lowest effective concentration required for control cells), the VIF network formed by vimC328N retracts into the perinuclear region in a fashion indistinguishable from controls, which express wild-type vimentin (Fig. 3B, D). Unfortunately, vimC328N does not work as a control, because it shows that the cysteine residue is not required for the WFA-induced reorganization of VIF networks.

### Withaferin A Alters the Phosphorylation State of Vimentin IF

**Figure 4:**
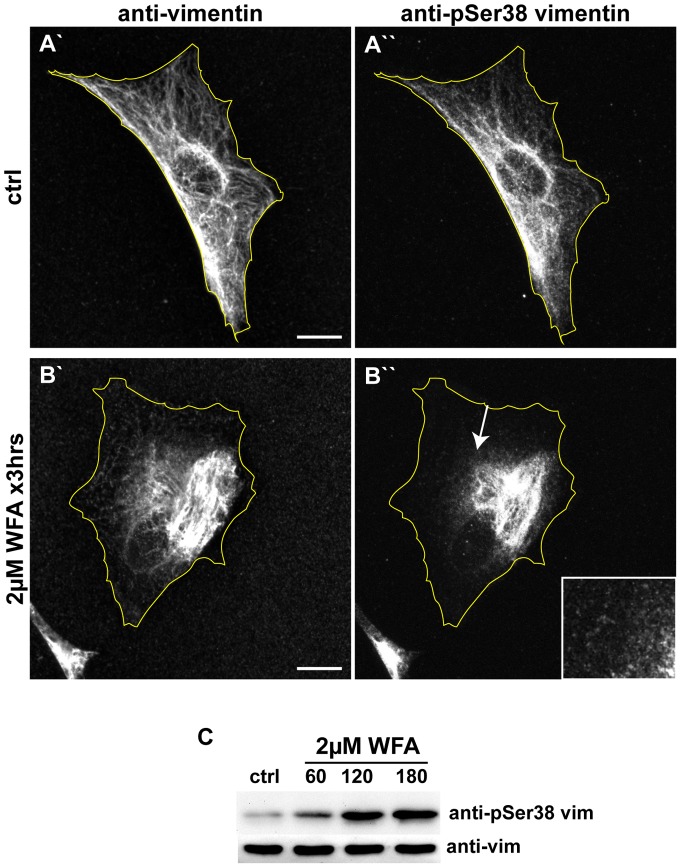
WFA treatment induces an increase in the phosphorylation of vimentin serine-38. BJ-5ta cells were treated for 3 hrs with DMSO (A) or 2 μM (B) WFA, then fixed and double labeled with vimentin (A′ and B′) and pSer38 vimentin (A′′ and B′′) antibodies. Scale bars =10 μm. Arrow: a region depicted at higher magnification in the inset showing vimentin particles stained with pSer38 vimentin antibody. (C) Whole cell lysates of cells treated with DMSO (ctrl) or 2 μM WFA for 60 min, 120 min, and 180 min, were separated by SDS-PAGE and stained with anti-vimentin and anti-vimentin pSer38 vimentin antibodies.

The phosphorylation state of VIF has been shown to affect their assembly states, cytoplasmic organization, and their dynamic properties [13], [19]. As indicated in the introduction, vimentin is phosphorylated at numerous sites [17] by different kinases [18]. Since serine-38 is a target for many of these kinases, it is a useful reporter of the phosphorylation state of VIF [18], [25]. Therefore, we determined the phosphorylation level of serine-38 before and after WFA treatment using an antibody specific for vimentin phosphorylated at this residue (pSer38, Fig. 4). Immunofluorescence shows that following treatment of BJ-5ta cells with 2 μM WFA for 3 hrs, pSer38 staining is observed in the retracted VIF located in the juxtanuclear region, as well as in particles and squiggles (Fig. 4B). Control cells contained a normal appearing distribution of VIF with both vimentin and pSer38 antibodies (Fig. 4A). The fluorescence intensity of VIF stained with the pSer38 antibody increased a small but statistically significant amount in WFA treated vs. control cells (from 1.45±005 in control cells to 1.48±008; SEM, n=25; p<0.05). In contrast, quantitative immunoblotting analyses of cell extracts prepared at 30, 60 and 180 min following exposure to 2 μM WFA showed a 2.5, 5.9, and 7.2 fold increase in vimentin phosphorylation, respectively, during WFA treatment (Fig. 4C). The discrepancy between the immunofluorescence and biochemical measurements may be explained by problems inherent in quantifying microscopic images including antibody accessibility and fixation protocol. However, there is little doubt based on the immunoblotting data that WFA treatment induces a significant increase in vimentin phosphorylation which is coincident with the juxtanuclear accumulation of VIF.

### Effects of Withaferin A on the Assembly of VIF *In Vitro*

**Figure 5:**
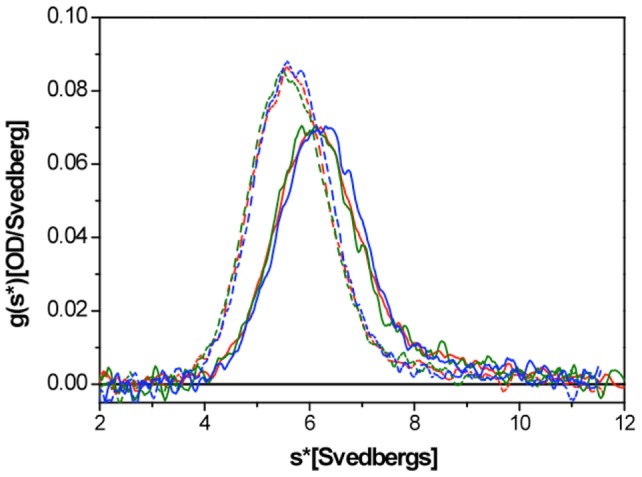
WFA does not affect the sedimentation velocity of vimentin. Sedimentation velocity profile of vimentin (0.15 mg/ml) reconstituted in 5 mM Tris-HCl, pH 8.4 (dashed lines) or 2 mM NaCl, pH 7.5 buffer (solid lines) alone (red lines), with DMSO (green lines), or with 25 μM WFA dissolved in DMSO (blue lines). Note that the curves of the two groups of runs are practically superimposable indicating that DMSO and DMSO plus WFA do not have any effect on the complex formation of vimentin oligomers.

Our results show that cysteine-328 is not required for the WFA induced reorganization of VIF in cells. However, *in vitro* studies clearly show that WFA binds to vimentin [2]. Based upon these findings we tested whether WFA alters any parameter of the polymerization of wild-type vimentin into IF *in vitro*. First, we analyzed the steps in assembly by sedimentation velocity ultracentrifugation in the presence and the absence of WFA (Fig. 5). We did this under two conditions previously demonstrated to provide optimal starter units for subsequent filament assembly: 5 mM Tris-HCl, pH 8.4 and 2 mM Na_3_PO_4_, pH 7.5 [28]. In both cases, neither DMSO alone nor WFA with DMSO had any influence on the sedimentation behavior of vimentin, which exhibited an *s*-value of 5.6 S (Tris-buffer) and 6.2 S (Na_3_PO_4_-buffer).

**Figure 6:**
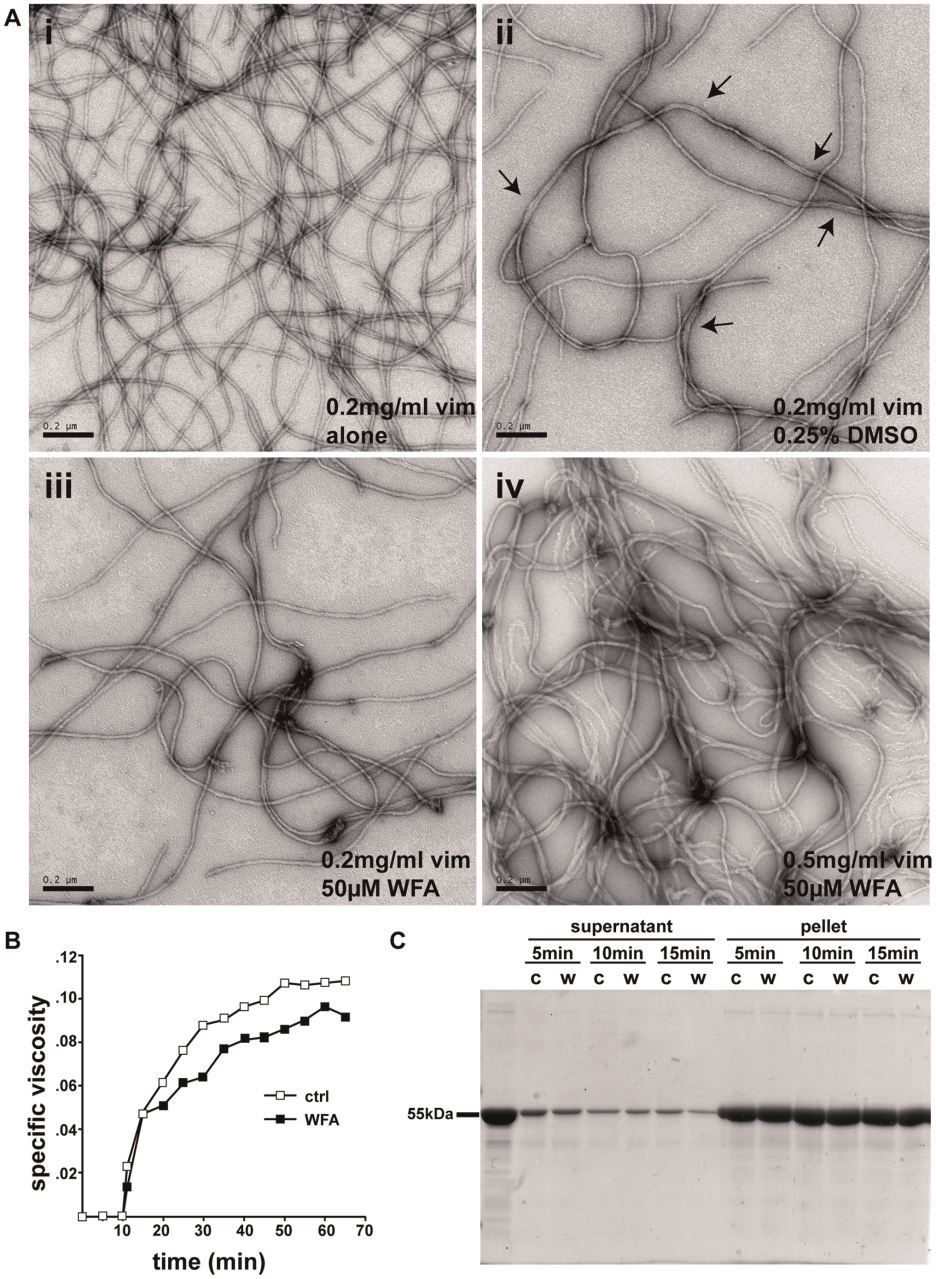
WFA has no effect on the *in vitro* assembly of human recombinant vimentin. (A) Recombinant human vimentin (0.2 mg/ml) was assembled for 10 min at 37°C in (i) 50 mM NaCl; (ii) with 0.25% DMSO; (iii) with 50 μM WFA; and (iv) for 30 min with 50 μM WFA at a protein concentration of 0.5 mg/ml. The filaments were fixed with glutaraldehyde and visualized by negative stain electron microscopy. The arrows in (ii) indicate lateral annealing and apparent fusion of individual filaments. (scale bars, 0.2 μm). (B) Viscometric analysis of vimentin assembly in the absence (ctrl) and presence of 50 μM WFA at 37°C in 50 mM NaCl. (C) Centrifugation assay of vimentin assembled in the absence (c) and presence of WFA (w). VIF were assembled for the indicated times (5 to 15 min) in 160 mM NaCl then centrifuged for 5 min at 10 psi in an Airfuge. Samples were separated by SDS-PAGE and stained with Coomassie. The position of vimentin is indicated (55 kDa).

The rate and extent of the assembly of VIF in the presence of WFA was observed by electron microscopy of negatively stained samples and viscometry as previously described [28]. In the presence of up to 50 μM WFA the structure of fully assembled VIF is not significantly altered compared to controls (compare Fig. 6A-i and Fig. 6A-ii to Fig. 6A-iii and Fig. 6A-iv). VIF appear uniform in length and width as observed by negative stain electron microscopy. Some VIF occasionally show lateral annealing, however, this effect is also seen in control samples (DMSO alone; Fig. 6A-ii, arrows). Furthermore, this DMSO-induced change is more obvious at higher protein concentrations. At 0.5 mg/ml protein (Fig. 6A-iv) and 1% DMSO (data not shown) VIF more often terminate in clump-like structures. Correspondingly, the behavior of VIF during viscometry and the rate of VIF assembly is only slightly altered by the addition of WFA (Fig. 6B), compatible with the electron microscopic data. Furthermore, VIF assembly also appears to be normal as determined by centrifugation/pelleting assays. Both with and without WFA, ∼100% of the vimentin is found in the pellet by 15 minutes following the initiation of assembly (Fig. 6C).

### Effects of Withaferin A on Other Types of Intermediate Filaments

**Figure 7:**
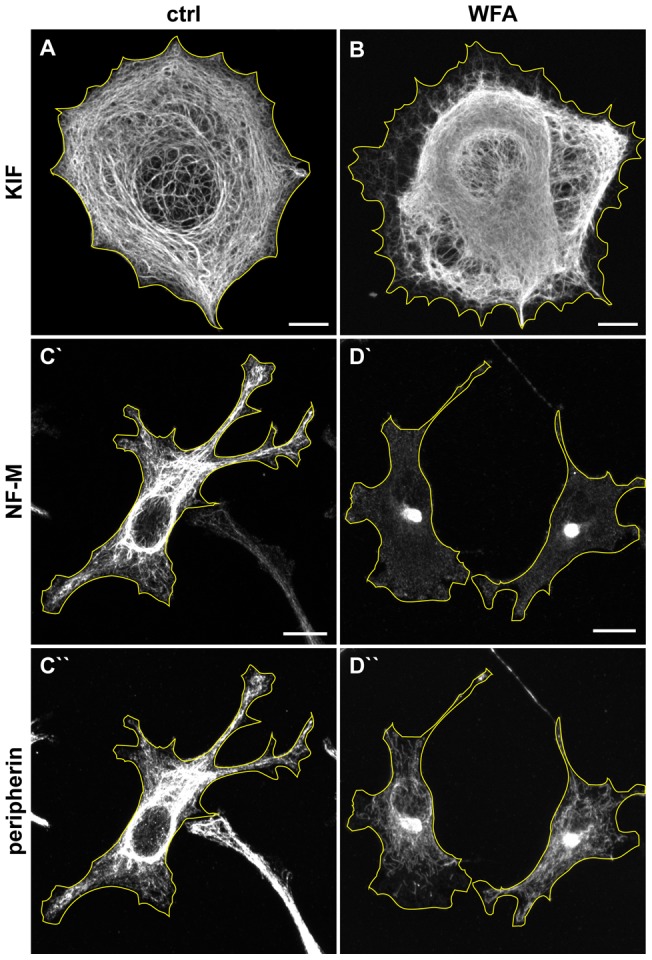
WFA affects the organization of keratin and neuron specific IF. MCF-7 cells, which express only keratin IF, were treated for 3 hrs with DMSO (A) and 4.0 μM WFA (B; the lowest effective concentration in these cells) and stained with pan-cytokeratin antibodies. Differentiated PC12 cells were treated for 3 hrs with DMSO (C) or 1.0 μM WFA (D, the lowest effective concentration for these cells), then stained with neurofilament-M (C′ and D′) and peripherin antibodies (C′′ and D′′). Scale bars =10 μm.

Different mammalian cells express one or more types of IF proteins. Since the proposed binding site for WFA is in a domain that is highly conserved in all cytoskeletal IF [11], we also determined whether WFA alters other types of IF networks. Therefore, we treated MCF7 epithelial cells, which express only types I and II keratin heteropolymer IF (KIF) with 1–3 μM WFA. No effects could be detected at these concentrations for 3 hrs. However, following treatment with 4 μM WFA for 3 hrs, the majority of the KIF network, which normally extends to the cell surface (Fig. 7A), retracts to form a large juxtanuclear aggregate (Fig. 7B). This response is similar to that of VIF in BJ-5ta cells. However, the reorganization is not as extensive as seen with VIF, as a subpopulation of KIF remain extended toward the cell periphery.

We also determined the effects of WFA on the type III IF comprised of peripherin found mainly in peripheral neurons [29], and type IV IF, containing neurofilament triplet proteins (NIF) present in the majority of neuronal cell types [30]. Pheochromocytoma cells (PC12) grown in nerve growth factor contain networks of peripherin IF (Fig. 7C'') and ∼50% also contain NIF (Fig. 7C') [31]. Treatment with 1 μM WFA for 3 hr induces the retraction of both peripherin IF and NIF to the nuclear region (Fig. 7D). Lower concentrations of WFA do not have a significant effect on either IF type over the same time period (not shown). The NIF form a clearly defined perinuclear cap, with few NIFs seen elsewhere in the cytoplasm (Fig. 7D′). Peripherin IF co-localize with NIF in the juxtanuclear aggregates, however, numerous peripherin structures also remain outside this cap (Fig. 7D′′). Based upon these observations it is apparent that WFA effects all four types of cytoskeletal IF.

### Insights into the Use of Withaferin A as an Anti-Cancer Agent

**Figure 8:**
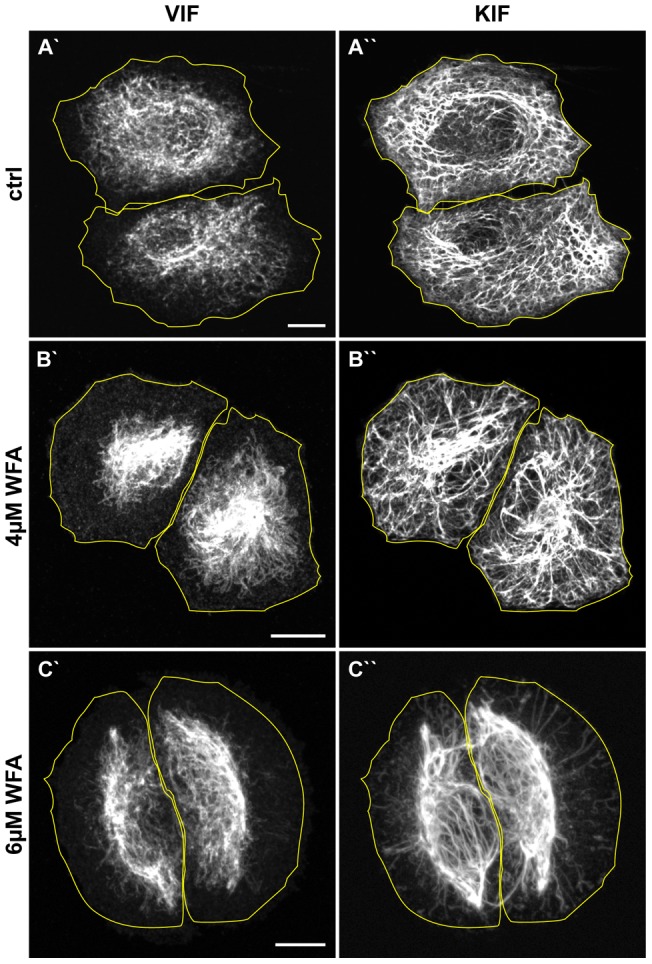
Keratin IF are less sensitive to WFA than VIF. Human lung cancer cells, A549, were treated for 3 hrs with DMSO [ctrl] (A), 4.0 μM WFA (B), and 6.0 μM WFA (C), followed by staining with vimentin (A′, B′, C′) and pan-cytokeratin antibodies (A′′, B′′, C′′). Scale bars =10 μm.

Most cancers arise from epithelial cells, which in their normal differentiated states express keratin (KIF), but not VIF. However, vimentin expression is up-regulated in, and is indeed one of the hallmarks of, the epithelial-mesenchymal transition (EMT) that accompanies the transformation of pre-metastatic to metastatic cells [3]. As a consequence, it is common for cancerous cells to express both VIF and KIF networks during the EMT. Based upon these findings, it has been proposed that specifically disrupting VIF using WFA in cells undergoing the EMT might prove useful in chemotherapy [4]. However, the proposed binding site for WFA in vimentin is highly conserved in all cytoskeletal IF. Therefore, it is important to determine whether WFA specifically disrupts VIF or whether it affects other types of IF as well. Thus, we determined the effects of WFA in human A549 lung cancer cells, which express both KIF and VIF (Fig. 8A). Treatment with a range of concentrations showed that after 3 hrs of exposure to 4 μM WFA, the VIF network reorganizes into the perinuclear region (Fig. 8B′), while KIF retain their normal distribution (Fig. 8B′′). Increasing the concentration of WFA to 6 μM induces the reorganization of both the VIF and KIF networks in 3 hrs (Fig. 8C). To determine whether the difference in sensitivity to WFA is attributable to a difference in the rate of retraction between KIF and VIF we treated the cells with 4 μM WFA for 6 hrs. Under these conditions the extent of reorganization of both IF networks is similar to that seen after 3 hrs (data not shown). Similar differences between VIF and KIF sensitivity to WFA are also observed in HeLa cells (data not shown).

**Figure 9:**
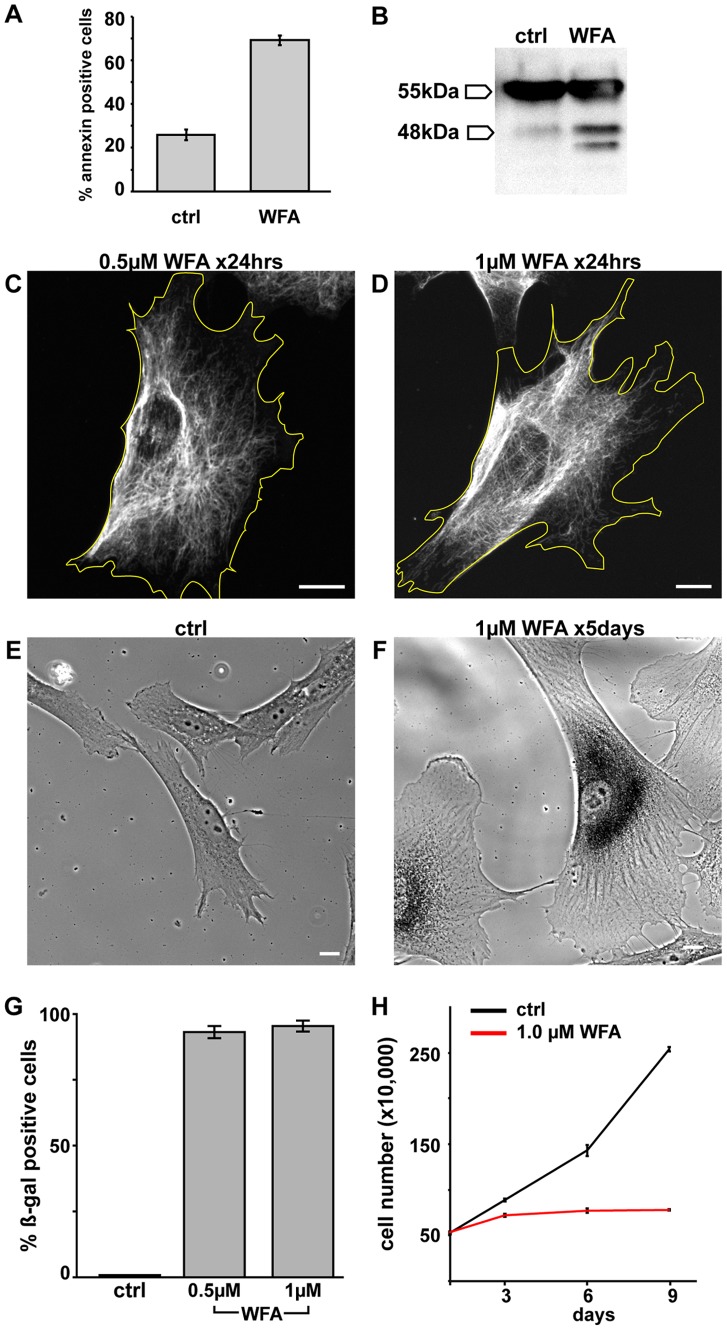
Longer exposure to WFA induces apoptosis or senescence. (A) BJ-5ta cells were treated for 24 hrs with DMSO and 2 μM WFA followed by staining with annexin V and assayed by FACS. (B) Whole cell lysates were separated by SDS-PAGE and blotted with anti-vimentin. BJ-5ta cells treated for 24 hrs with0.5 μM WFA (C) and1 μM WFA (D) were stained with vimentin antibodies. BJ-5ta fibroblasts were incubated with DMSO alone (E) or 1 μM WFA (F) continuously for 5 days and stained for senescence-associated ß-galactosidase. (H) Cell proliferation of BJ-5ta cells was monitored by counting cells every 3 days during continuous incubation with DMSO alone (black line) or 1.0 μM WFA (red line). Scale bars =10 μm.

We also examined the effects of long-term treatment of cells with WFA as this would be required in cancer treatment. To this end, BJ-5ta cells were treated with 2 μM WFA for 24 hrs. Following this period of exposure, 76.1%±3.2% of the cells die as determined by Trypan blue exclusion. We also determined that apoptosis is the most likely cause of cell death as 69.0±2.2% of the cells treated with WFA are positive for annexin V [32]; compared to 25.8±2.4% in control cells (Fig. 9A). Induction of apoptosis by WFA is confirmed by immunoblotting for the caspase-dependent breakdown of vimentin to a 48 kDa fragment (Fig. 9B) [33].

Cells treated with lower concentrations of WFA (0.5 μM and 1 μM) for 24 hrs remain viable and contain less extensively altered VIF networks as indicated by a slight retraction of VIF away from the cell periphery (compare Fig. 1A to Fig. 9C and D). Following 5 days of exposure to 0.5 μM or 1 μM WFA, many cells appear larger (compare Fig. 9 E to F, data for cell exposed to 0.5 μM not shown), suggesting that they are senescent. In support of this, the majority of cells are positive for senescent associated β-galactosidase after exposure to 0.5 μM or 1 μM WFA (93.1%±2.3%, n=158 and 95.4%±2.1% n=131, respectively) compared to 0.47%±0.81% (n=655) in control cells (Fig. 9 G).

Based upon these findings, we also determined the rate of BJ-5ta cell proliferation by monitoring the number of cells every 3 days over a nine-day period. The rate of cell proliferation is similar between treated cells and controls during the first 3 days of WFA treatment at a concentration of 1.0 μM WFA. However there is a dramatic decrease in growth rate after day 3 in the WFA treated cells (Fig. 9F). These data show that relatively low concentrations of WFA, that do not dramatically reorganize VIF, arrest the growth of cells by inducing senescence.

### Withaferin A Also Affects the Organization of Microtubules and Microfilaments

**Figure 10:**
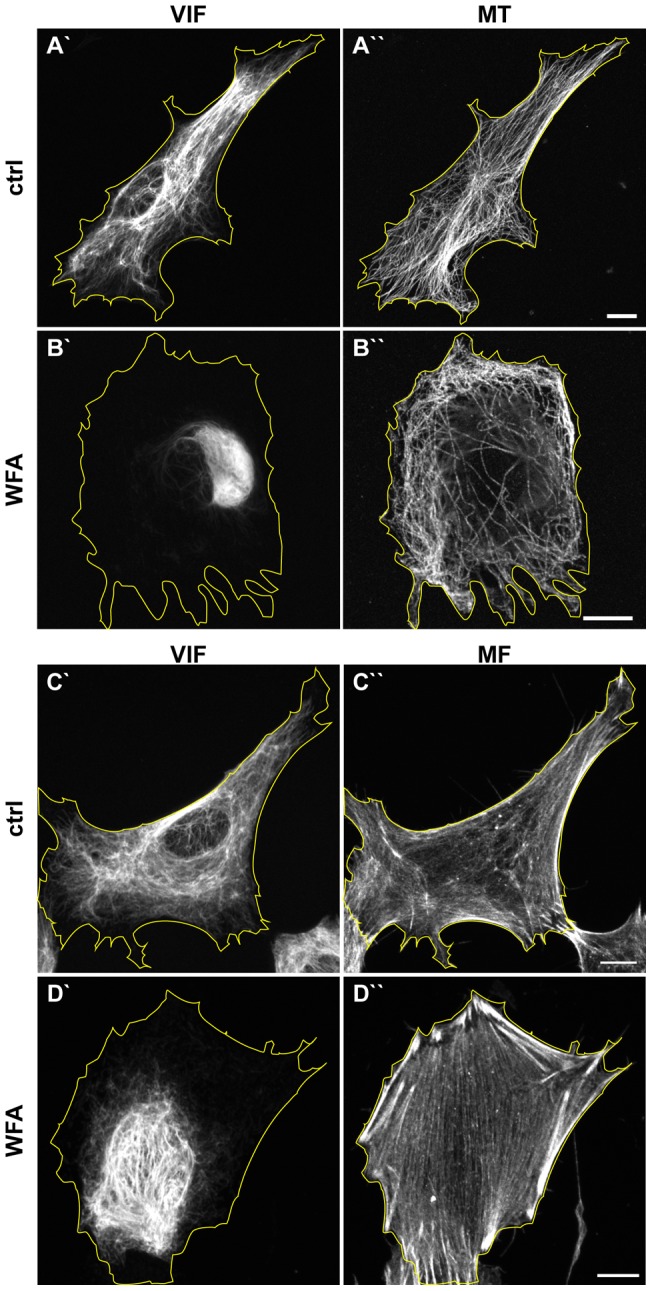
WFA also alters the organization of microtubules and microfilaments. BJ-5ta cell were treated with DMSO (A and C) and 2 μM WFA (B and D) for 3 hrs and stained with vimentin antibodies (A′, B′, C′, D′), tubulin antibodies (A′′ and B′′), and phalloidin to visualize to actin (C′′ and D′′). Scale bars =10 μm.

Since IF interact extensively with microtubules (MT), and actin/microfilaments (MF) [19], we determined whether WFA alters these cytoskeletal systems. In control BJ-5ta cells, normal arrays of MTs are seen radiating from a centrally located microtubule-organizing center (MTOC) near the nucleus (Fig. 10A′′). Following 3 hrs of exposure to 2 μM WFA, VIF are reorganized into a perinuclear aggregate (Fig. 10B′). There appear to be fewer MTs at the cell center compared to controls and these are distributed mainly toward the cell periphery. In addition, the MTOC is not apparent in 89.2%±3% (n=615) of the cells compared to 2%±1% (n=600) in controls (compare Figs 10A′′ and B′′). Following WFA treatment many MTs appear wavy, rather than straight as seen in controls (compare Fig. 10A′′ and B′′). Under the same conditions of WFA treatment there is an apparent increase in the number of actin stress fibers and the appearance of extensive “sheets” of actin not observed in control cells (compare Figs 10 C′′ and D′′).

## Discussion

Withaferin A causes the redistribution of VIF in fibroblasts, from their normal arrays extending throughout the cytoplasm, into perinuclear aggregates. Reorganization of VIF into juxtanuclear aggregates has also been reported in cells exposed to either microtubule depolymerizing agents such colchicine [34], or microtubule stabilizing drugs such as Taxol [35]. Other conditions that induce aggregates include the expression of dominant negative vimentin mutants [36], [37] and the elevated phosphorylation of vimentin in response to the overexpression or microinjection of kinases such as PKA [38], PKC, and CaM kinase II [39]. The loss of normal VIF organization is often accompanied by changes in cell shape and motility. For example, micro-injection of a mimetic peptide drives the specific disassembly of VIF and causes fibroblasts to round up [26]. Similarly, there are changes in cell shape and a reduction of migration when vimentin expression is down-regulated by shRNA [24]. Based upon these and other observations, it is not surprising that the aggregation of VIF induced by WFA is accompanied by changes in cell shape and motility. The effects of WFA on motility may be related to the role that VIF have been shown to play in regulating the formation of lamellipodia. A gradient of vimentin assembly states from non-filamentous particles, short IF, to long IF has been observed in moving fibroblasts [25]. Fully polymerized VIF along the cell periphery appear to prevent the membrane from ruffling and forming lamellipodia. In contrast, the disassembly and retraction of these peripheral VIF induces the formation of lamellipodia. Therefore, WFA may inhibit cell motility by disrupting the organization of VIF that is required for normal cell locomotion.

*In vitro* studies suggest that WFA binds covalently to cysteine-328 [2]. However, when a mutant of vimentin lacking cysteine-328 is expressed in cells null for vimentin, it assembles into networks which appear normal and respond to WFA in a manner indistinguishable from the response of wild-type VIF. Since there is evidence that WFA binds to vimentin [2], even though this does not appear to require cysteine-328, we tested whether WFA can affect assembly of VIF *in vitro*. However we could not detect any alteration in the assembly kinetics of vimentin nor in the formation of their tetramer building blocks. Overall our results demonstrate that there are no detectable changes in the polymerization of vimentin subunits into 10 nm IF in the presence of WFA. Although it would be interesting to determine whether WFA binds to VIF in cells, this would require a method for labeling and localizing this small molecule in fixed cells or *in vivo*. Unfortunately this methodology is not yet available. Overall our data suggest that WFA may interact with other factors involved in regulating IF organization such as kinases or phosphatases. Support for this comes from the finding that WFA treatment is associated with a significant increase in phosphorylation of vimentin at serine-38. This particular residue is targeted by at least seven different protein kinases including PKA, PKC, Rho-Kinase, and Akt [14], [18]. Therefore, it is possible that the increase in phosphorylation of vimentin reflects a WFA-induced modulation in the activity of one of the kinases or phosphatases that regulate the assembly state and subcellular organization of VIF [13], [38].

The changes in VIF networks in the presence of WFA are accompanied by alterations in the organization of MT and MF. This is not surprising as VIF and MTs have been shown to interact either directly [40]; or indirectly via a number of factors including the microtubule based motors, kinesin [41], [42] and dynein [43]; and/or other IF and microtubule associated proteins such as plectin [44] and MAP2 [45]. There is also evidence that actin interacts directly with vimentin [21] and indirectly through the IF associated protein, plectin [22]. Hence, it is clear that IF interact with both MTs and MFs and that a perturbation of any one of these three cytoskeletal systems leads to the disruption of the other two systems.

In addition to VIF, WFA also alters the organization of peripherin, keratin, and neurofilament IF. Previous studies have demonstrated that the distribution of networks comprised of glial fibrillary acid protein (GFAP), another type III IF, is also altered during WFA treatment [46]. In our studies to date we have not been able to detect obvious alterations in the organization of the Type V IF composed of the lamins, which are exclusively localized in the nucleus (data not shown). Therefore, WFA is not specific to VIF but can alter a variety of cytoskeletal IF systems.

Prior to metastasis epithelial derived tumor cells undergo an epithelial to mesenchymal transition (EMT). One of the hallmarks of the EMT is an induction of vimentin expression [3]. In light of this, several investigators have used WFA to selectively target cells that express vimentin. In line with our findings that WFA inhibits cellular migration and proliferation, WFA reduces tumor growth, invasion, and metastasis in animal models [3], [4], [8]. Additionally, since vimentin is the major IF type in endothelial cells [47] and probably the most abundant cytoskeletal protein in these cells (unpublished observations), it is not surprising that WFA also inhibits angiogenesis [4], [8], [9]. However, in light of our finding that WFA induces the aggregation of other types of IF that are prevalent in many different types of tissues, a thorough examination of the ancillary effects of WFA is warranted. Support for possible side effects comes from the finding that numerous diseases are characterized by aggregates of IF that are frequently located in the perinuclear region. These include amyotrophic lateral sclerosis [48], Alzheimer's disease [49], [50], diabetes [51], and giant axonal neuropathy [52]. Therefore, it is conceivable that treatment with WFA may cause aggregation of IF and subsequent dysfunction of multiple tissue types. On the other hand, our finding that different IF types exhibit different sensitivities to WFA suggests that it may be possible to titrate its concentration so that it primarily affects metastatic cancer cells while not disrupting the function of normal tissue.

## Materials and Methods

### Cell culture

Human BJ-5ta fibroblasts (ATCC, Manassas, VA) and A549 (ATCC, Manassas, VA) cells were maintained in DMEM (Invitrogen, Carlsbad, CA) supplemented with 10% fetal calf serum (FCS, HyClone, Logan, UT). SW13-1HF5 [53] cells were grown in Leibovitz L-15 (Invitrogen, Carlsbad, CA) medium supplemented with 10% FCS. MCF7 cells (ATCC, Manassas, VA) were maintained in MEM (Invitrogen, Carlsbad, CA) supplemented with 10% FCS, 5 mM non-essential amino acids (Cellgro, Manassas, VA), 1 mM sodium pyruvate (Cellgro, Manassas, VA), and 2 mM L-glutamate (Invitrogen, Carlsbad, CA). PC12 cells (ATCC, Manassas, VA) were maintained in DMEM with 10% calf serum (CS, HyClone, Logan, UT) and differentiated by changing the growth medium to DMEM supplemented with 5% CS and 50 ng/ml nerve growth factor as previously described [31]. All cells were grown in a humidified CO_2_ incubator at 37°C as described [12].

### Drugs

For cell based experiments withaferin A (WFA) was purchased from ChromaDex (Irvine, CA) and dissolved in DMSO (Sigma, St. Louis, MO) at a stock concentration of 10 mM. WFA was added to culture medium at different concentrations for varying time intervals. Controls consisted of adding DMSO to culture medium at the same dilutions.

### cDNA Constructs and Transfection

Construction of wild-type vimentin expression plasmid is described elsewhere [54]. Vimentin C328N mutant cDNA was generated by site-directed mutagenesis (Quickchange, Stratagene, Germany). Cells were transfected by electroporation as described previously [55].

### Antibodies

Vimentin IF were stained with either mouse monoclonal anti-vimentin V9 (Sigma, St. Lois, MO) or in Fig. 10A and B with chicken polyclonal anti-vimentin (Covance, Princeton, NJ). Other antibodies used were: rat polyclonal anti-yeast α-tubulin (Sigma, St. Louis, MO), mouse polyclonal anti-pan-cytokeratin (Sigma, St. Louis, MO), rat monoclonal anti-pSer38 vimentin TM38 [56], rabbit polyclonal anti-peripherin #199 [57], and mouse monoclonal anti-neurofilament 160 (Sigma, St. Louis, MO). Actin was labeled by staining with phalloidin conjugated to Alexa568 (Invitrogen, Carlsbad, CA). Secondary antibodies included Alexa488-, Alexa568- conjugated goat anti-rabbit, anti-mouse, anti-chicken, and anti-rat IgG (Jackson ImmunoResearch, West Grove, PA).

### Immunofluorescence

Cells grown on glass coverslips were rinsed with phosphate buffered saline (PBS) and fixed in methanol at −20°C for 5 min. For Figure 10 C and D cells were fixed in 3.7% formaldehyde for 10 min at room temperature to preserve actin. Subsequently, the cells were incubated with the appropriate primary antibody for 30 min at 37°C in a humidified chamber. After three washes with PBS they were incubated with appropriate secondary antibodies for 30 min at 37°C. The coverslips were then mounted on glass slides in 100 mM Tris pH 9.0, 50% glycerol (v/v) containing 2 mg/ml p-phenylenediamine (Sigma, St. Louis, MO). Fluorescence images of fixed/stained cells were taken using a Zeiss Confocal LSM 510 microscope equipped with a Plan-Apochromat 1.4NA 63x objective (Carl Zeiss, Jena, Germany). The cell periphery was traced using phase contrast images and superimposed over fluorescent images using Adobe Photoshop CS5 (San Jose, CA). The mean fluorescence intensity was calculated by importing images into Volocity 3D Image Analysis Software (PerkinElmer, Waltham, MA), thresholding the image, subtracting background pixels, and calculating the mean object intensity for each image and channel. All scale bars are 10 μm except where noted.

### Live Cell Imaging

Time-lapse observations of live cells were made using the Nikon Eclipse TE2000-E microscope equipped with the Perfect Focus system (Nikon, Meliville, NY) and an INU stage incubator system (Tokai Hit, Shizuoka-Ken, Japan). Images were captured using a CoolSnapEZ camera (Photometrics, Tucson, AZ) at 5 min intervals through a 10x objective.

### Cell Motility Assay

The rate of cell motility was determined by tracking the position of the center of the nucleus over time using Metamorph v7.0 (Molecular Devices, Sunnyvale, CA). The distance between the positions were determined and divided by the interval between the time points and then averaged. The motility of control cells was determined for one hour in normal growth medium. Cell motility was monitored for 4 hrs in 2 μM WFA; the medium was refreshed and motility was monitored for an additional 16 hrs.

### Cell Shape

For quantification of cell shape, fixed cells were imaged using a Zeiss AxioImager.Z1 equipped with a 40x phase contrast objective and a Zeiss AxioCam MRm camera (Carl Zeiss, Jeta, Germany). Cell perimeter and area were determined by tracing the cell perimeter using Zeiss AxioVision software (Carl Zeiss, Jeta, Germany). Cell shape was expressed using form factor (FF), 4π*(area)/(perimeter)∧2, as described previously (Soll, 1988).

### Senescence Assay

The induction of cellular senescence by treatment with WFA was determined by staining for β-galactosidase activity according to previously published protocols [58]. Briefly, cells grown on glass coverslips were fixed with 3.7% formaldehyde, 0.2% glutaraldehyde for 10 min at room temperature. After thorough rinsing, the cells were incubated for 4 hrs at 37°C with 50 μg/ml X-gal, 8 mM sodium citrate pH 6.0, 5 mM potassium ferrocyanide, 5 mM potassium ferricyanide, 150 mM NaCl, and 2 mM MgCl_2_.

### Apoptosis Assay

Cells were stained for annexin V externalization using the Annexin V Apoptosis Assay (Clontech, Mountain View, CA) according to the manufacturer's instructions and counted by FACS.

### Analysis of the Effects of WFA on the Assembly of Vimentin IF *In Vitro*

Recombinant human vimentin, solubilized in 8 M urea, was renatured by overnight dialysis into either 5 mM Tris-HCl (pH 8.4) or 2 mM sodium phosphate (pH 7.5) containing 1 mM DTT as described [28]. For analytical ultracentrifugation the samples were dialyzed for one hour against fresh buffer without DTT. Sedimentation velocity runs were performed exactly as described [28]. Withaferin A (99% pure by HPLC, Enzo Life Sciences, Farmingdale, NY) was dissolved in DMSO (20 mM) and further diluted such that vimentin solutions contained final concentrations of 25 μM or 50 μM WFA and 0.25 to 1.0% DMSO, respectively. The drug was pre-incubated with re-natured vimentin for 1 to 30 min at 37°C prior to initiation of assembly. Assembly was carried out in the Tris-buffer system; it was initiated by raising the ionic strength to 160 mM NaCl for 5 to 15 min as indicated [28]. For viscometry, the protein concentration was 0.5 mg/ml; for electron microscopy of negatively stained samples it was routinely 0.2 mg/ml except for the control for the viscometric analysis where it was 0.5 mg/ml. In this case the sample was fixed with 0.1% glutaraldehyde and diluted 1∶5 with assembly buffer before application to the grid. The extent of assembly was controlled by centrifugation using an Airfuge® (Beckman Coulter, Fullerton, California). The samples were spun for 5 min at 10 psi, the supernatant removed and complemented with half the volume of three times (3x) concentrated SDS sample buffer; the pelleted filaments were resuspended in an equal volume of SDS sample buffer containing 6 M urea. Samples were heated for 3 min at 95°C and run on 10% polyacrylamide gels according to the procedure of Laemmli.

### Statistics

Quantitative results are presented as mean ± standard deviation from three separate experiments. P-values were determined by a two-tailed Students t-test with unequal variances. Results were considered significant when the p-value was equal or less than 0.05.
